# Extraction of polysaccharides from Maca enhances the treatment effect of 5-FU by regulating CD4^+^T cells

**DOI:** 10.1016/j.heliyon.2023.e16495

**Published:** 2023-05-24

**Authors:** Fenghua Cao, Hanyuan Zhang, Ying Yan, Yi Chang, Jie Ma

**Affiliations:** a Zhenjiang Hospital of Chinese Traditional and Western Medicine, Zhenjiang 212000, China; b Department of Laboratory Medicine, School of Medicine, Jiangsu University, Zhenjiang 212013, China

**Keywords:** Maca polysaccharide, CD4^+^T cells, 5-FU, Immunoregulatory

## Abstract

In our previous studies, we used a graded alcohol precipitation method to extract four maca polysaccharide components (MCP1, MCP2, MCP3, and MCP4) from maca with various molecular weights. Compared to other three components, MCP2 had stronger immunoregulatory abilities on CD4^+^T cells. To avoid the immunosuppressive effect of 5-fluorouracil (5-FU), maca polysaccharides in combination with 5-FU treatment were investigated in this study. The results show that 500 mg/kg and 1000 mg/kg MCP2 could significantly delay the growth of tumor and enhance the anti-tumor effect of 5-FU *in vivo*. Furthermore, MCP2 can partly recover the proliferation of CD4^+^T cells after being suppressed by 5-FU *in vitro*. Additionally, in order to explore the mechanism in which MCP2 acts on CD4^+^T cells, the MCP2 is marked with FITC fluorescence and synthesis MCP2-Tyr-FITC for the first time. Confocal microscope results show that MCP2-Tyr-FITC can directly bind to the surface of CD4^+^T cells. Together, our work demonstrates that maca polysaccharides could enhance the anti-tumor effect when combined with 5-FU by regulating CD4^+^T cells, suggesting a novel potential immunomodulator in tumor therapy.

## Graphical abstract

**Figure:**
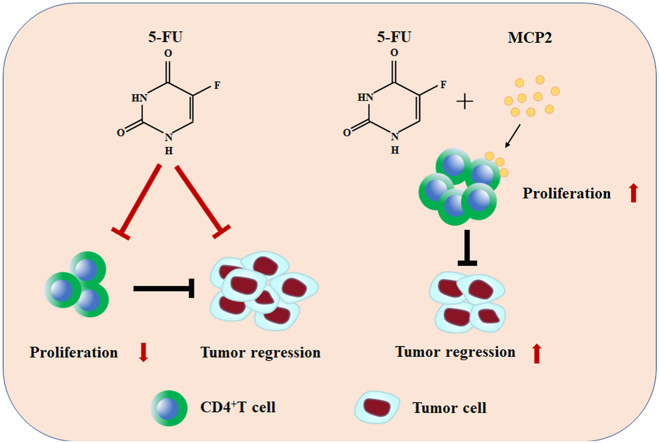

## 1 Introduction

Maca (*Lepidium meyenii*) is a kind of plant native to the Andean region of central Peru. It has been cultivated for at least 2000 years and generally grows at an altitude of 4000 m characterized by extreme weather conditions, such as rocky soil, intense sunlight, and a windy atmosphere [1]. Maca root contains high nutritional value, such as protein (10–18%), carbohydrates (59–76%), a variety of amino acids, trace elements and minerals, as well as trace amounts of active ingredients such as polysaccharides, macamides, macaenes, and alkaloids [[2], [3], [4]]. In recent years, Maca has been widely used as a dietary supplement because of its pharmacological activities, such as improving fertility, improving memory, anti-fatigue, anti-depressant and anti-oxidation [[5], [6], [7], [8], [9]].

As an important component of Maca, Maca polysaccharides (MP) are composed of galactose, rhamnose, arabinose, glucose, xylose, fucose, mannose, and so on [10]. It is worth noting that studies on the immunomodulatory effect of Maca polysaccharides are mostly about macrophages [11]. Lee et al. reported that MP could inhibit lipopolysaccharide-induced NO production in RAW 264.7 cells, which indicated that an increasing concentration of MP enhanced anti-inflammatory activities [12]. Yang et al. reported subsequently that MP had a strong tumor-associated macrophage remodeling effect, suggesting that it may be a promising biomaterial for cancer immunotherapy [13]. In addition, Maca polysaccharides have been reported which also affect intestinal epithelial cells and nerve cells. MC-1 and MC-2, two polysaccharides were identified in the roots of maca, activated intestinal immunity and alleviated intestinal inflammation by regulating intestinal epithelial cells [14]. MP protected neuronal cells from oxidative stress through a mechanism including a decrease in lactate dehydrogenase (LDH) leakage and reversal of H_2_O_2_-induced cell morphological damage [15]. Furthermore, MP is involved in immune regulation by activating T lymphocytes. The mechanism was related to inhibiting lymphocyte apoptosis and promoting the balance of Th1/Th2 cell subsets [16]. Our previous research had confirmed that MCPs (MCP1, MCP2, MCP3, and MCP4) had the function of promoting CD4^+^T cell proliferation and interferon-γ (IFN-γ) secretion, especially MCP2 [17].

5-Fluorouracil (5-FU) is a commonly used chemotherapeutic drug used in the treatment of various malignancies, but chemotherapy damages the patient's immune system while killing tumor cells. In recent years, scholars have done a lot of exploration, hoping to find a more effective form of adjuvant chemotherapy with fewer side effects. Polysaccharides have always been one of the hot spots in the field. Studies have shown that there are many kinds of polysaccharides that have anti-tumor and anti-oxidant effects. HPW, which is a bioactive polysaccharide derived from insects, had protective effects on the intestinal mucosa and could relieve intestinal inflammation caused by drug side effects [18]. Polysaccharides extracted from *Albuca bracteata Thunb., J.C. Manning, and David Goldblatt (ABP)* exhibited anti-neoplastic activity and could effectively enhance the efficacy of 5-FU in CRC treatment [19].

In this study, we investigated the immunomodulatory effects and mechanisms of MCP2, one of the components of Maca polysaccharides, combined with 5-FU on tumor growth and immune function in tumor-bearing mouse.

## 2 Materials and methods

### 2.1 Maca polysaccharides

Dried Maca was purchased from Qinghai Shennong Biotechnology Co., Ltd. The color of dried maca was yellow. The dried maca was cut into pieces and ground into a powder. After being sieved through a 200-mesh screen, the powder was stored in a tightly closed, dry container. The details of the preparation of Maca polysaccharides are in reference [19].

### 2.2 Preparation of Maca polysaccharides

As mentioned in our previous research [17], four fractions were obtained, labeled MCP1, MCP2, MCP3, and MCP4 respectively.

### 2.2 5-FU

The 5-FU (25 mg/ml) was purchased from Tianjin Jinyao Amino Acids Co., Ltd. (Tianjin, China) under batch number 0812291.

### 2.3 Cell line, mice and tumor model

The Lewis lung carcinoma (LLC) cells were obtained from the cell bank of the Shanghai Institutes for Biological Sciences. C57BL/6 mice (6–8 weeks old, 18–22 g, male) were purchased from the Animal Research Center of Jiangsu University (Zhenjiang, China) and housed in a pathogen-free environment. Mice were implanted subcutaneously (s.c.) with LLC cells (5 × 10^5^ cells/mouse) to construct the xenograft model. The experimental protocols were approved by the Institutional Committee on the Use of Animals for Research and Teaching.

### 2.4 Tumor weight and volume

After one week of adaptation to the environment, the mice were weighed every 3 days after the first gavage treatment. After the tumor is tumor-bearing, the tumor size is monitored daily. After the mouse can touch the size of the soy bean tumor (accessible on the fifth day after tumor-bearing), the tumor volume is measured every 3 days until the mice are sacrificed.).The volume of each tumor was calculated by the formula: tumor volume = 1/2 ab^2^, where ‘a’ is the largest diameter and ‘b’ is the perpendicular diameter [18].

### 2.5 Isolation of murine CD4^+^T cells

As mentioned in our previous research [17], the isolation of murine CD4^+^T cells was performed according to the manufacturer's protocol of CD4 MicroBeads (Milteny Biotec).

### 2.6 CD4^+^T cell proliferation assay

CD4^+^T cells were sorted from wild type C57BL/6 mice using CD4 microbeads (Miltenyi Biotec), labeled with carboxyfluorescein succinimidyl amino ester (CFSE) using the CellTraceTM CFSE Cell Proliferation kit (Invitrogen, Carlsbad, CA, USA), and were co-cultured with different ratios of 5-FU and maca polysaccharides in U-bottomed 96-well plates (Costar) in the presence of 10 μg/ml anti-CD3 mAb (Biolegend) and 5 μg/ml anti-CD28 mAb (Biolegend) for 72 h. The control group contained only CD4^+^T cells activated with anti-mouse CD3e and CD28 functional grades.

### 2.7 Fluorescence of maca polysaccharide

Weighed Maca polysaccharide (MCP2) at 400 mg and dissolved in 20 ml double steamed water. The pH of the solution was adjusted with NaHCO_3_ to 8.0. Added 400 mg of tyrosine and 150 mg to the polysaccharide solution and mixed it. After the reaction system was placed on the shaking bed for 96 h, supernatant was collected. Added the supernatant to the Sephadex G-200 columns, collected the first peak of the flowing, scanned with an ultraviolet lighting meter in the range of 200–600 nm, frozen and dried to get the MCP2-Tyr.

Dissolved MCP2-Tyr obtained above in 20 ml of double-steamed water, and adjusted the pH with NaHCO_3_ to 8.5. Added FITC (30 mg) to react, kept at room temperature and dark place for 12 h. After the reaction was over, the water-free ethanol was added o the ethanol, which now makes up 75% of the total volume. At this time, yellow-green precipitation will be precipitated. Centrifuge at 1000 rpm for 5 min and collect the precipitation. The collected precipitation was dissolved in 20 ml of double-steamed water and repeatedly sunk three times to remove excess fluorescein Isothiocyanate (FITC). The powder obtained after freezing precipitation is crude MCP2-Tyr-FITC.

### 2.8 The purification of MCP2-Tyr-FITC

Put the Sephadex G-200 gel expanded in excessively double-steamed water for 3 h at room temperature. Added the inflatable gels to the column, then opened the constant-current pump, so that the double steamed water could flow at a speed of 0.7 ml/min. The dry MCP2-Tyr-FITC powder weighed 20 mg and was dissolved in 2 ml of dual-steamed water. After filtering through a 0.45 μm liquid-phase filter membrane, the polysaccharide solution was added to the Sephadex G-200 column; the flow rate was set to 0.5 ml/min; and a tube was collected every 5 min, for a total of 30 tubes. The collected solution was detected by a phenol-sulfuric acid method to detect the polysaccharide content of each tube collection liquid. Then detected the fluorescent at 520 nm of each tube by the fluorescence spectrophotometer. Drew the polysaccharide-fluorescent curve, collected the components with the largest sugar content and fluorescent strength, and frozen and dried them to get MCP2-Tyr-FITC.

### 2.9 Measurement of the fluorescent substitution efficiency of MCP2-Tyr-FITC

The measurement of the fluorescent substitution efficiency of MCP2-Tyr-FITC was carried out according to reference [20]. I dissolved 0.8 mg of FITC in 1 ml of dual-steamed water, diluted the 100 μL solution to 10 ml, and this was a FITC solution of 8 μg/ml. Then, we diluted this solution to obtain a solution of 4.0, 2.0, 1.0, 0.5, and 0.25 μg/ml in order, and detected the fluorescence intensity of each tube. Drew the standard curve, using the concentration of FITC as the horizontal coordinate and the fluorescence intensity as the vertical coordinate. Dissolve 1.0 mg MCP2-Tyr-FITC in 5 ml of dual-steamed water accurately, diluted 0.5 ml MCP2-Tyr-FITC solution to 2 ml and detected the fluorescent intensity. The quality of the FITC contained in the 100 μg of MCP2-Tyr-FITC powder represented the fluorescent substitution efficiency.

### 2.10 Observe the combination of MCP2-Tyr-FITC and CD4^+^T cells by laser confocal microscope (LSCM)

Added MCP2-Tyr-FITC with a final concentration of 50 μg/ml to the cell culture system, and the bottom of the plate was pre-placed with a cap of 12 mm in diameter. After 48 h of cultivation, the cultivation board was centrifuged and the supernatant was discarded. Added PBS solution containing PE-CD4 antibodies in cell culture system for 30 min in 4 °C, then added PBS solution and placed on the shaking bed for 5 min, repeating 3 times. Used 4% polymerization-formaldehyde to fix the cells overnight, the next morning, washed and removed the polymerized formaldehyde solution by PBS. Dyed the nucleus with DAPI solution, sealed the glass slice with paraffin oil, and observed by a laser confocal microscope (LSCM).

### 2.11 Statistical analysis

Data are expressed as mean ± SD. The statistical significance of differences between groups was determined by ANOVA using SPSS 19.0 software. Data from all experiments was imported into GraphPad Prism 5.0 to generate bar graphs. ***p < 0.005, **p < 0.01, *p < 0.05, ns (no significance).

## 3 Results

### 3.1 MCP2 combined with 5-FU treatment delayed the growth of tumor

**Fig. 1:**
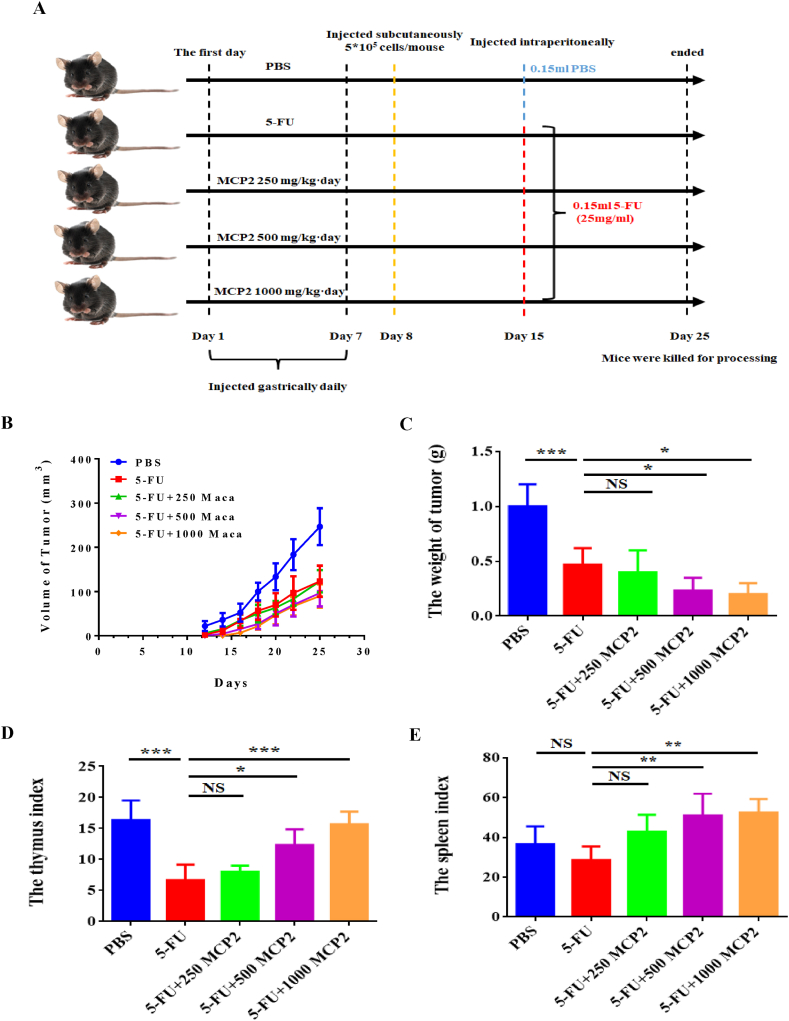
Combination of MCP2 and 5-FU exerted a stronger anti-tumor effect than 5-FU alone. A: The establishment of tumor-bearing mice model, including PBS group, 5-FU group, 5-FU+250MCP2 group, 5-FU+500MCP2 group, and 5-FU+1000MCP2 group; B: The volume of tumors; C: The weight of tumors; D The thymus index of different groups; E: The spleen index of different groups, *p < 0.05, **p < 0.01, ***p < 0.001, NS: no significant differences.

30 mice were adapted to the environment for a week before the experiment. Mice were implanted subcutaneously (s.c.) with LLC cells (5 × 10^5^ cells/mouse) to construct the xenograft model. After that, the mice were randomly separated into 5 experimental groups: PBS (PBS only), 5-FU (5-FU treatment); 5-FU+250 MCP2 (250 mg/kg/day MCP2+5-FU), 5-FU+500 MCP2 (500 mg/kg/day MCP2+5-FU), and 5-FU+1000 MCP2 (1000 mg/kg/day MCP2+5-FU). Each group had six mice and was treated as shown in Fig. 1A. Measured the tumor volume every two days. The mice were sacrificed on Day 25, and the tumor tissues were dissected and weighed. The results showed that compared to the PBS group, the tumor volume of the 5-FU group was significantly decreased, MCP2 combined with 5-FU group even lower, and there was a certain concentration-dependent manner (Fig. 1B). The tumor weight also showed the same trend as tumor volume. Compared with the 5-FU group, the 5-FU treatment group assisted with Maca polysaccharides had a reduced tumor weight (Fig. 1C). The thymus index and the spleen index were calculated, the results showed that the thymus index of the PBS group was significantly higher than that of the 5-FU group, and that MCP2 could recover the thymus index after 5-FU treatment. The spleen index of the PBS, 5-FU, 5-FU+250 MCP2, 5-FU+500 MCP2 and 5-FU+1000 MCP2 groups was 16.33 ± 1.856, 6.667 ± 1.453, 8.000 ± 0.5774, 12.33 ± 1.453 and 15.67 ± 1.202, respectively (Fig. 1D). The thymus index of PBS, 5-FU, 5-FU+250 MCP2, 5-FU+500 MCP2 and 5-FU+1000 MCP2 was 36.5 ± 5.220, 28.5 ± 3.984, 42.73 ± 4.967, 50.90 ± 6.414 and 52.47 ± 3.875 individually (Fig. 1E). The results showed that MCP2 could partially restore the decreasing thymus index caused by 5-FU. Together, the above results indicated that Maca polysaccharides could enhance the anti-tumor activity of 5-FU.

### 3.2 MCP2 increased the number and percentage of CD4^+^T cells in 5-FU treatment mice

**Fig. 2:**
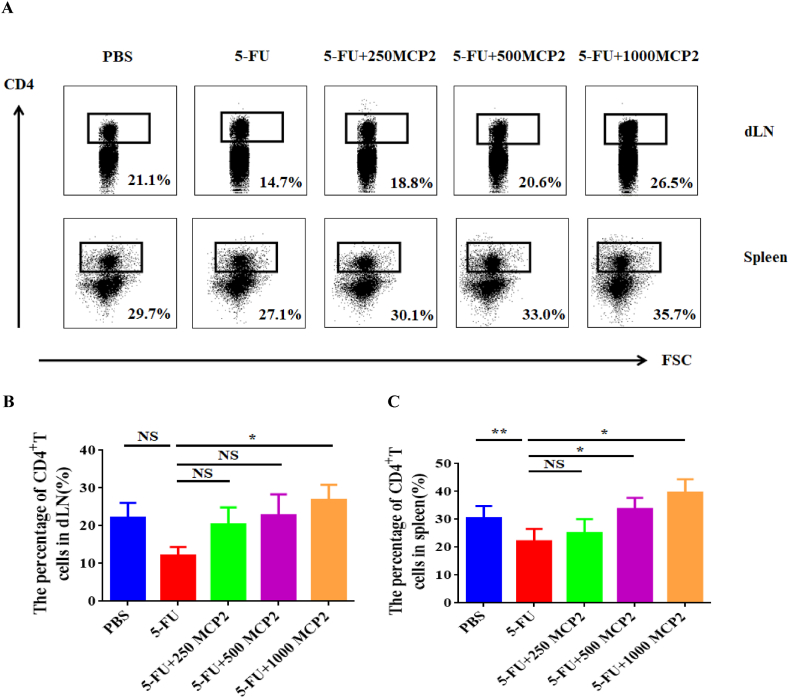
MCP2 increased the percentage of CD4^+^T cells both in spleen and dLN of tumor-bearing mice. A: Typical flow cytometry of percentage of CD4^+^T cells; B: The percentage of CD4^+^T cells in lymph nodes; C: The percentage of CD4^+^T cells in spleen, *p < 0.05, **p < 0.01, NS: no significant differences. dLN: draining lymph node.

After sacrificing the tumor-bearing mice, the spleen was made into single-cell suspension, CD4^+^T cells were isolated and purified by immunomagnetic beads *in vitro*, so that purity could meet the requirements of subsequent experiments. The typical flow cytometry for the percentage of CD4^+^T cells is shown in Fig. 2A. Compared with mice treated with PBS, the results showed that in the mice treated with 5-FU, the numbers and frequencies of CD4^+^T cells were significantly decreased in the spleen. When MCP2 was used as an auxiliary treatment with 5-FU, we could find the percentage of CD4^+^T cells in the spleen and draining lymph node (dLN) had a rising tendency, and there was a certain concentration dependence. When the concentration of MCP2 reached 1000 mg/kg, the percentage of CD4^+^T cells in dLN was significantly increased (Fig. 2B), and when the concentration of MCP2 reached 500 mg/kg, the percentage of CD4^+^T cells in the spleen also significantly increased compared with the 5-FU group (Fig. 2C). Above results indicated that 5-FU destroyed the immune environment of the body and reduced the proportion of CD4^+^T cells both in the spleen and the dLN, while Maca polysaccharides could reduce the inhibitory effect of 5-FU on CD4^+^T cells in chemotherapy, and the concentration was most obvious when the concentration reached 1000 mg/kg.

### 3.3 MCP2 partially restored the suppression of CD4^+^T proliferation caused by 5-FU *in vitro*

**Fig. 3:**
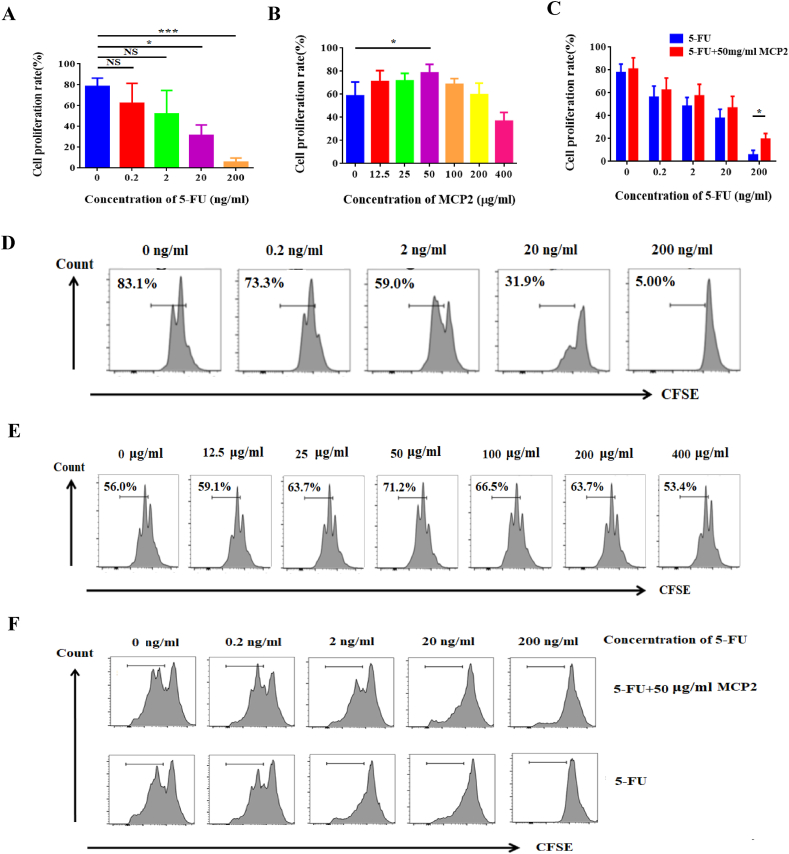
MCP2 partly recovered the proliferation of CD4^+^T cells inhibiting by 5-FU. A: The CD4^+^T cells proliferation rate of different concentration of 5-FU (0, 0.2, 2, 20. 200 ng/ml); B: The CD4^+^ T cells proliferation rate of different concentration of MCP2 (0, 12.5, 25, 50, 100, 200, 400 μg/ml); C: The proliferation of CD4^+^T cells treated with different concentrations of 5-FU (0, 0.2, 2, 20. 200 ng/ml) and 50 μg/ml MCP2 for 72 h; D–F: Typical flow cytometry of proliferation of CD4^+^T cells. *p < 0.05, ***p < 0.001, NS: no significant differences.

After sacrificing the tumor-bearing mice, CD4^+^T cells of the spleen were separated and purified by immune magnetic beads, and the purity of CD4^+^T cells was 96.0%. Firstly, we observed the inhibition of 5-FU on the proliferation of CD4^+^T cells. The sorted CD4^+^T cells were cultured with different concentrations of 5-FU (0, 0.2, 2, 20, 200 ng/ml) for 72 h and cell proliferation was detected by CFSE. The results showed that with the increased concentration of 5-FU, the proliferation rate of CD4^+^T cells was decreased. When the concentration of 5-FU reached 20 ng/ml, the proliferation efficiency of CD4^+^T cells decreased significantly compared with the control group (0 ng/ml) (Fig. 3A,D). 5-FU could inhibit the proliferation of CD4^+^T cells *in vitro*.

Secondly, we explored the effect of MCP2 on the proliferation of CD4^+^T cells. The sorted CD4^+^T cells were co-cultured with different concentration of MCP2 (0, 12.5, 25, 50, 100, 200, and 400 μg/ml) for 72 h. As in Fig. 3B and E, the proliferation rate of CD4^+^ T cells rose first 50 μg/ml of MCP2 was the highest point, and then began to decline.

Finally, in order to further investigate whether Maca polysaccharides could restore the growth of CD4^+^T cells caused by 5-FU, we used 50 μg/ml MCP2 to assist with 5-FU *in vitro*. The results showed that 50 μg/ml of MCP2 could restore the proliferation rate of CD4^+^T cells significantly at a concentration of 20 and 200 ng/ml of 5-FU (Fig. 3C,F). In conclusion, because the results of the *in vitro* experiments were similar to those of the *in vivo* experiments, MCP2 could enhance the therapy effect of 5-FU by promoting the proliferation of CD4^+^T cells.

### 3.4 Preparation and purification of MCP2-Tyr-FITC

**Fig. 4:**
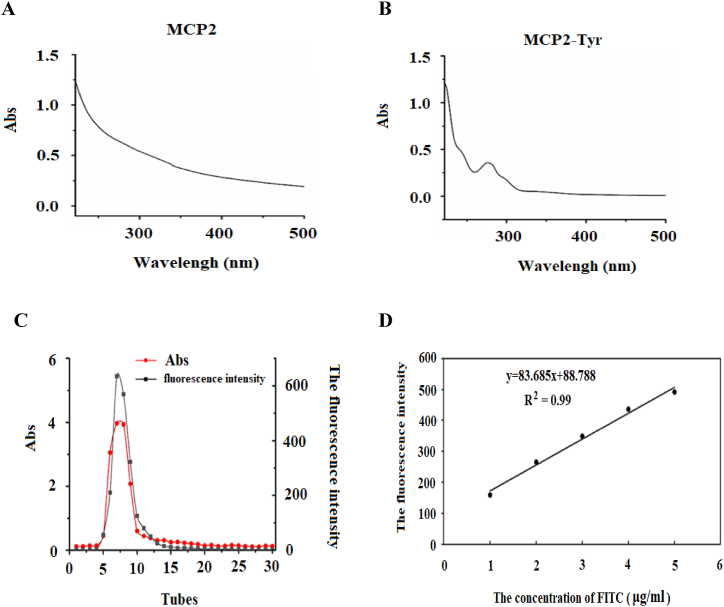
Prepared MCP2-Tyr-FITC successfully. A: UV spectrum of MCP2 solution; B: UV spectrum of MCP2-Tyr solution; C: Elution curve of MCP2-Tyr-FITC Sephadex G-200 gel column; D: Standard curve of FITC content.

Now that we h know that Maca polysaccharide could regulate CD4^+^T cells, we are interested in the mechanism. In order to observe whether the MCP2 would combine with the surface of the activated CD4^+^T cells or it would be swallowed by the cells, we tried to label the MCP2 with FITC. The MCP2 was first dissolved and reacted with tyramine and sodium borohydride for 72 h, after which the supernatant was added to the chromatographic column, the first peak of the flow was collected, and the peak was detected using an ultraviolet lighting meter. As in Fig. 4A, there was no obvious absorption peak at 280 nm, which meant MCP2 did not react with sodium and sodium borohydride. If an obvious absorption peak appeared at 280 nm, indicating that MCP2 had successfully reacted with the sodium and sodium borohydride (Fig. 4B). After centrifuging the flowing sample, they frozen and dried the supernatant, and that was MCP2-Tyr.

Secondly, dissolved the MCP2-Tyr, which obtained from above, and FITC together for 12 h, then added the water-free ethanol. The yellow-green precipitation would be observed. Collected and dissolved the Yellow-green precipitation and removed the FITC, which did not combine with polysaccharides. The precipitation was dissolved, frozen, and dried to obtain a crude MCP2-Tyr-FITC. Dissolved the crude MCP2-Tyr-FITC and added it to the Sephadex G-200 gel column to collect flow for 30 tubes. The fluorescence intensity of each tube was detected by a fluorescence photometer. As shown in Fig. 4C, the polysaccharide content and FITC content of the 7th and 8th tubes were the highest among all the tubes. Therefore, the outflow of the 7th and 8th tubes was collected as purified MCP2-Tyr-FITC.

Finally, we drew the standard curve of FITC and calculated the fluorescent substitution efficiency of MCP2-Tyr-FITC to be 4.9%, according to the fluorescent intensity of MCP2-Tyr-FITC.

### 3.5 MCP2-Tyr-FITC combined CD4^+^T cells on membrane surface

**Fig. 5:**
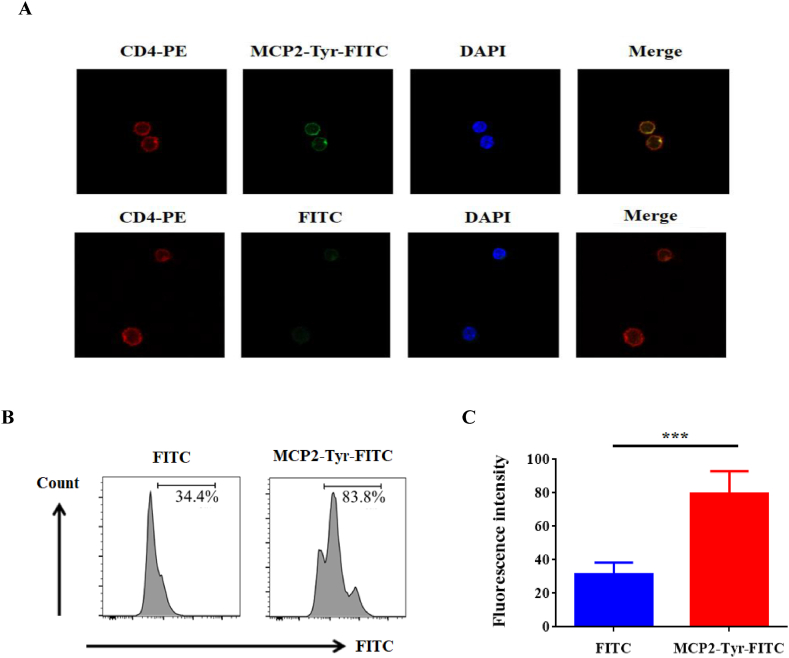
MCP2 binds to CD4^+^T cells. A: Confocal microscope observation effect of FITC and MCP2-Tyr-FITC on CD4^+^T cells (red: PE-CD4 antibody; green: MCP2-Tyr-FITC; blue: DAPI); B: Typical flow diagram of the fluorescence intensity of CD4^+^T cells after treatment of FITC and MCP2-Tyr-FITC respectively; C: The statistical figure of the fluorescence intensity of CD4^+^T cells treatment of FITC and MCP2-Tyr-FITC respectively, ***p < 0.001. (For interpretation of the references to color in this figure legend, the reader is referred to the Web version of this article.)

Added FITC and MCP2-Tyr-FITC into the culture system of CD4^+^T cells respectively. Collected the cultured cells and observed them by laser confocal microscopy after 48 h. The results showed that compared to the CD4^+^T cell of the FITC group, the CD4^+^T cell of the MCP2-Tyr-FITC group had much brighter green fluorescence (Fig. 5A). After merging the image, the results indicated that green fluorescence and red fluorescence almost overlapped, indicating that MCP2-Tyr-FITC was combined on the membrane surface of CD4^+^T cells.

In order to come to the above conclusion, we also detected the fluorescent intensity on the cell surface of CD4^+^T cells by FCM. Fig. 5B is the typical FCM image of fluorescent intensity detection. The fluorescent intensity on the membrane surface of CD4^+^T cells in the MCP2-Tyr-FITC group was significantly higher than that of CD4^+^ T cells in the FITC group (Fig. 5C). The above results confirmed again that MCP2-Tyr-FITC could combine on the membrane surface of CD4^+^T cells.

## 4 Discussion

5-FU, as one of the most commonly used chemotherapy drugs, kills the tumor cells directly, but in this process, it also has a negative effect on normal cells, including immune cells. Polysaccharides are a type of natural active product, and some have been shown to have immunomodulatory and anti-tumor properties. The mode of the anti-tumor activity of polysaccharides is totally two-fold: one way is to kill tumor cells directly such as polysaccharides from Phaeodactylum tricornutum [21], and the other way is to play a synergistic and attenuating role in chemotherapy such as Lentinan [22]. Some research has reported that Maca polysaccharides could assist in the treatment of tumors [23,24].

Our previous research has also confirmed that all four extracts of Maca polysaccharides have the effect of promoting CD4^+^T cell proliferation, especially MCP2 [17]. In this study, we want to further clarify the function and mechanism of MCP2 in tumor therapy. We found that the combination of MCP2 and 5-FU exerted a stronger anti-tumor effect than 5-FU alone while delaying the growth of tumors. The function of immunomodulation in the body depends on the normal function of immune organs, immune cells, and immune factors. The index and spleen index can directly reflect the level of immune functions in the body [25]. In this study, compared to treatment with 5-FU alone, after it was combined with MCP2, the number and proportion of CD4^+^T cells, the thymus index and the spleen index of tumor-bearing mice were all greatly increased. We also found the similar results during *in vitro* experiment. We focused on the CD4^+^T cells and found out that Maca polysaccharides could partly recover the proliferation of CD4^+^T cells inhibited by 5-FU. These experiments provide some experimental evidence for the further clinical application of Maca polysaccharides.

Some publications have reported the regulating mechanism of polysaccharides on immune cells, especially T cells. Orally administered Alhagi honey polysaccharide could remarkably induce T (CD4^+^ and CD8^+^) cell and B cell proliferation and activation [26]. Wen-Ting Fei et al. reported that the mechanism of immunomodulatory effect of Maca polysaccharides was related to inhibiting lymphocyte apoptosis and promoting the balance of Th1/Th2 cell subsets [16]. Taraxasterol regulated the immunity of H22 bearing mice by elevating the ratio of CD4^+^T cells in the spleen, and increasing the number of T cell infiltration in tumor tissue [27]. But due to the lack of easily detectable light-emitting groups, further research on polysaccharides has always been hindered. Wanrong Bao et al. connected FITC to RAP, and observed that FITC-RAP could directly combine with hematopoietic stem cells (HSC) in bone marrow [28]. So, on the basis of the above work, we tried to synthesize MCP2-Tyr-FITC as probes in order to further clarify and the mechanism of polysaccharides on CD4^+^T cells. In this study, we synthesized MCP2-Tyr-FITC, and according to the standard curve of FITC fluorescent intensity, the fluorescent replacement degree of MCP2-Tyr-FITC is 4.9%. MCP2-Tyr-FITC and FITC were added to the CD4^+^T cell culture system, respectively. The results showed that MCP2-Tyr-FITC could combine the membrane surfaces of CD4^+^T cells. On this basis, we will further explore the binding site between MCP2-Tyr-FITC and CD4^+^T cells in the future.

The anti-tumor pathway of polysaccharides is mainly through regulating the immune system. Usually, polysaccharide can bind to related receptors on the surface of immune cells, such as toll-like receptors (TLRs), mannose receptors (MR), anti-beta glucan Receptor (Dectin-1), scavenger receptors (SR), and complement receptor 3 (CR3), activate downstream signal pathways, and regulate the immune system to play an immunomodulatory role [29,30]. Our previous research also investigated the effect of particulate β-glucans on myeloid-derived suppressor cells (MDSCs) via the dectin-1 pathway *in vitro* [30]. Interestingly, we found that maca polysaccharide can bind to the surface of CD4^+^T cells, but the specific receptor needs further study and confirmation. After binding to receptors on the cell surface, maca polysaccharides may activate intracellular signal transduction pathways, and mediate inflammatory factors and other substances are released, thereby enhancing the ability of immune regulation.

In this study, MCP2 was obtained from Maca, and the immunomodulatory effect on a xenograft model in combination with 5-FU was studied. The results showed that MCP2 could improve the general state of the model, promote the proliferation of CD4^+^T cells, and increase the thymus index and the spleen index. All these data indicate that MCP2 has potent immunomodulatory properties and might be considered a novel potential immunomodulator for use in drugs or functional foods. Furthermore, this study successfully combined FITC with Maca polysaccharides for the first time, which lays the foundation for the fluorescence mark of polysaccharides, and the in-depth research on the mechanism of polysaccharides.

## Author contribution statement

Fenghua Cao, Ying Yan: Performed the experiments; Analyzed and interpreted the data; Wrote the paper.

Hanyuan Zhang, Jie Ma: Conceived and designed the experiments.

Yi Chang: Performed the experiments; Analyzed and interpreted the data.

## Funding statement

Fenghua Cao was supported 10.13039/501100013094Science and Technology Planning Social Development Project of Zhenjiang City {SH2021027}, Clinical Medical Science and Technology Development Foundation of 10.13039/501100002703Jiangsu University {JLY2021149}.

## Data availability statement

Data included in article/supp. material/referenced in article.

## Declaration of competing interest

The authors declare that they have no known competing financial interests or personal relationships that could have appeared to influence the work reported in this paper.

## List of abbreviations

5-FU: 5-Fluorouracil
MP: Maca polysaccharides
LDH: lactate dehydrogenase
IFN-γ: interferon-γ
LLC: The Lewis lung carcinoma
CFSE: carboxyfluorescein succinimidyl amino ester
FITC: fluoresceinIsothiocyanate
LSCM: laser confocal microscope
dLN: draining lymph node
TLRs: Toll-like receptors
MR: mannose receptors
Dectin-1: anti-beta Glucan Receptor
SR: scavenger receptors
CR3: complement receptor 3
MDSCs: myeloid-derived suppressor cells
